# Consideration on the Intergenerational Ethics on Uranium Waste Disposal

**DOI:** 10.1007/s40572-024-00442-x

**Published:** 2024-03-28

**Authors:** Hiroshi Yasuda, Hiromichi Fumoto, Tatsuo Saito, Shin-etsu Sugawara, Shoji Tsuchida

**Affiliations:** 1 Research Institute for Radiation Biology and Medicine, Hiroshima University, 1 Kasumi 2-3, Minami-ku, Hiroshima, Japan; 2 Japan Inspection Co. Ltd., 2–9–1 Hatchobori, Chuo-ku, Tokyo, 104–0032 Japan; 3 4-49 Muramatsu, Tokai-mura, Ibaraki, Naka-gun 319-1112 Japan; 4 Faculty of Societal Safety Sciences, Kansai University, Hakubai-cho 7-1, Takatsuki, Osaka, 569-1098 Japan

**Keywords:** Uranium waste, Radioactive, Disposal, Intergenerational ethics, Responsibility

## Abstract

**Purpose of Review:**

This review provides insights into resolving intergenerational issues related to the disposal of waste containing high amounts of uranium (uranium waste), from which distant future generations will have higher health risks than the current generation.

**Recent Findings:**

Uranium (half-life: 4.5 billion years) produces various progeny radionuclides through radioactive decay over the long term, and its radioactivity, as the sum of its contributions, continues to increase for more than 100,000 years. In contrast to high-level radioactive wastes, protective measures, such as attenuation of radiation and confinement of radionuclides from the disposal facility, cannot work effectively for uranium waste. Thus, additional considerations from the perspective of intergenerational ethics are needed in the strategy for uranium waste disposal.

**Summary:**

The current generation, which has benefited from the use and disposal of uranium waste, is responsible for protecting future generations from the potential risk of buried uranium beyond the lifetime of a disposal facility. Fulfilling this responsibility means making more creative efforts to convey critical information on buried materials to the distant future to ensure that future generations can properly take measures to reduce the harm by themselves in response to changing circumstances including people’s values.

## Background

### Uranium Wastes

Utilization of nuclear power accompanied by uranium purification, conversion, enrichment, reconversion, and fabrication inevitably generates a waste containing concentrated uranium (uranium waste). The amount of uranium waste is predicted to grow along with the expansion of nuclear power. Uranium waste is comprised of residues, equipment, filter cores, resins, etc., which contain low-level uranium isotopes existing mostly in the form of U_3_O_8_, UO_2_, and UF_6_.

Radioactive wastes are classified into several types of radioactive waste, as shown in Table 1 [1, 2]. The method of radioactive waste disposal has been determined in accordance with the radioactivity and half-lives of the major radionuclides contained in the waste. For example, the high-level radioactive waste (HLW), which contains a large amount of fission products (^137^Cs, ^90^Sr, etc.) is to be disposed of deep underground in the form of vitrified wastes. On the other hand, low-level radioactive waste (LLW) with relatively low radioactivity generated from hospitals and industrial/research facilities as well as nuclear power plants will be disposed of at shallow depths from the ground surface. However, as shown in Table 1, there is currently no international consensus on the strategy for uranium waste disposal. Accordingly, in many countries, uranium wastes are temporarily stored in interim storage facilities on-site.

**Table 1:** Classifications of radioactive wastes [1, 2]

Type	Origin	Material feature	Radioactivity
High-level waste (HLW)	Operation of nuclear facilities, fuel reprocessing, decommissioning	Spent nuclear fuel, vitrified reprocessed liquid waste	10^4^ to 10^6^ TBq m^-3^, 0.06% worldwide waste volume and 95% of the total radioactivity
Intermediate-level waste (ILW)	Operation and decommissioning of nuclear facilities, fuel reprocessing, nuclear medicine	Fuel cladding, end pieces, resins, sludge, clean up liquid of reactor coolant, spare parts, etc.	3.7 × 10^5^ to 3.7 × 10^8^ Bq g^-1^, 1.63% worldwide waste volume 3% of the total radioactivity
Low-level waste (LLW)	Operation and decommissioning of nuclear facilities, nuclear medicine, research, industry	Contaminated paper, rags, tools, clothing, injectors, filters, patient urine, stool, etc.	< 3.7 × 10^5^ Bq g^-1^, 69% worldwide waste volume 1.5% of the total radioactivity
Very low-level waste (VLLW)	Operation and decommissioning of nuclear facility, industry	NORM/TENORM from the mining and processing of ores, and minerals	< few ten Bq g^-1^, 29% worldwide waste volume 0.5% of the total radioactivity
Very short-lived waste (VSLW)	Nuclear medicine, research, industry	Short half-life radionuclides (^99m^Tc, ^131^I, ^192^Ir, etc.)	Shortly falling beneath the clearance level (i.e., moving to EW)
Exempt waste (EW)	Background radiation, soil, granite, rocks, and minerals	All kinds of radionuclides	With the dose rate of < 0.01 mSv y^-1^

One reason for the difficulty in classifying uranium waste is that uranium isotopes have extremely long half-lives, whereas their concentrations are generally low. All isotopes of uranium are radioactive, and this element is characterized by long-lived radionuclides such as ^238^U (half-life: 4.47 × 10^9^ years; mass percentage: 99.5%; specific activity: 1.25 × 10^4^ Bq g^-1^), ^235^U (7.04 × 10^8^ years; 0.72%; 8.0 × 10^4^ Bq g^-1^), and ^234^U (2.46 × 10^5^ years, 0.006%, 2.31 × 10^8^ Bq g^-1^) [3]. Of these uranium isotopes, only ^235^U is fissile and used as a fuel for nuclear power generation. ^238^U, the most abundant isotope of uranium, has the longest half-life of 4.5 billion years. Whereas the weight percentage of ^234^U is notably small, this isotope contributes as much as ^238^U to the total radioactivity because of its higher specific activity. Additionally, uranium generates various progeny radionuclides through the radioactive decay process; for example, ^238^U decays to radium (^226^Ra), radon (^222^Rn), polonium (^214^Po, ^210^Po), bismuth (^214^Bi, ^210^Bi), and so on, until it becomes a stable lead (^206^Pb) [4].

Although there has been a long debate in Western countries and international organizations regarding the categories in which uranium waste should be managed and disposed of, there are currently no internationally common guidelines. While the International Atomic Energy Agency (IAEA) has set the regulatory value of activity concentration for disposing radionuclides of natural origin as 1 Bq g^−1^ [5], no clear classification of uranium waste has been presented [1, 6]. Under this circumstance, selected countries have established their own general policies for managing and disposing uranium waste. For example, in the United States, uranium waste is treated differently from low-level radioactive waste, whereas uranium slag is classified as radioactive waste in a different way from that for the by-product of ordinary nuclear industry waste [7]. In the UK, while the Committee for Radioactive Waste Management (CoRWM) has suggested the inclusion of depleted, natural, and low-enriched uranium (DNLEU) in its recommendations for geological disposal [8], the UK Nuclear Decommissioning Authority (NDA) has stated that near-surface disposal of DNLEU is possible, although feasibility should depend on the characteristics of the disposal site [9]. In several other countries, residues containing high concentrations of uranium are stored as potentially usable resources and their disposal is an issue for future consideration [10].

Another concern is that uranium is a heavy metal that is chemically toxic. It has been reported that uranium intake can cause a variety of adverse health effects by impairing the kidneys, bones, liver, brain, lungs, and reproductive system [11–15]. While the bone acts as an initial reservoir of uranium absorbed by the human body, damage to the kidney is of primary concern as a high level of uranium accumulates in the renal tissue in the process of elimination through urine [11]. In addition, uranium can cross the blood-brain barrier and accumulate in the brain, causing neurological disorders [15]. Chronic exposure to uranium is thought to induce subclinical illnesses, such as hypertension, increased carcinogenesis, and cognitive decline, as seen in lead intake [14]. However, research on the chemotoxicity of uranium have been relatively slow compared to its radiotoxicity, and thus, more efforts to clarify its health risks and underlying toxicological mechanisms are required.

### Principles and Ethical Values of Radiological Protection

The difficulty in developing the strategy for uranium waste disposal is partially attributable to the fact that uranium is essentially a naturally occurring element [16]. In fact, uranium waste is generated solely due to a change in the abundance of uranium isotopes through the enrichment process. Thus, there has been a question about treating uranium waste in the same manner as other radioactive waste containing artificially generated radionuclides under the framework of the current system of radiological protection [17••].

In many countries including Japan, regulations related to radiological protection are in line with the basic recommendations of the International Commission on Radiological Protection (ICRP) [18, 19]. The ICRP has indicated the following three fundamental principles in its latest recommendations (Publication 103) [19]:

- Justification, which states that any decision which alters the exposure situation should do more good than harm.
- Optimization of protection, which stipulates that all exposures should be kept as low as reasonably achievable, taking into account economic and societal factors.
- Application of dose limits, which declares that individual exposures should not exceed the dose limits recommended by ICRP.

The 2^nd^ principle of optimization is also called the “ALARA (as low as reasonably achievable)” concept. Following the last principle on dose limits, the ICRP has recommended specific individual dose limits of 1 mSv y^-1^ for the general public, 100 mSv every 5 years, and 50 mSv per year for workers. Additionally, ICRP has recommended a dose constraint of 0.3 mSv y^-1^ for potential exposure of the public from radioactive waste disposed of [19, 20••].

The ICRP classifies situations in which people are exposed to radiation into three categories [19]:

- Planned exposure situations, which involve the introduction and operation of radiation sources.
- Emergency exposure situations, which indicates unexpected conditions that may occur during the operation under a planned situation or from a malicious act, requiring urgent attentions.
- Existing exposure situations, which already exist when a decision on the control of radiological exposure must be made, such as those caused by natural background radiation sources.

The principles of justification and optimization apply to all three exposure situations whereas the principle of application of dose limits applies only to planned exposure situations. For emergencies and existing exposure situations, reference levels higher than dose limits or dose constraints are recommended. However, regarding uranium waste disposal, it is still unclear which exposure situation is applied to the potential exposure of distant future generations.

In a recent publication on ethical foundations of the system of radiological protection (Publ. 138) [21•], the ICRP stated that the current system has been underpinned by four core ethical values: beneficence/non-maleficence, prudence, justice, and human dignity. These core values were followed by procedural values, which came into play for the practical application of the system: accountability, transparency, and inclusiveness. Among these core values, dignity is an attribute of the human condition, which means that each individual deserves unconditional respect, regardless of their age, sex, state of health, social condition, ethnic origin, and/or religion. This value of dignity should apply to future generations that are yet to be born. Relevant concepts have been presented also by the United Nations (UN); for example, the Universal Declaration of Human Rights stated “All human beings are born free and equal in dignity and rights” [22], and the UN’s sustainable development goals (SDGs), a critical conceptual basis of right actions for our generation, include the responsibility of current generations towards future generations [23].

Under these circumstances, the authors shared thoughts that it is important to discuss the issues of uranium waste disposal from the perspective of humanities and social science and then voluntarily organized a study group in the Japan Health Physics Society (JHPS) in 2020 [24]. In several meetings of the study group, the authors had a broad range of discussions for 2 years with many participants including some of the Japanese members of ICRP task groups and employees of electric power companies who had interests in deepening the understanding of the ethical aspects of the underground disposal of uranium waste. At that time, concepts and methods for uranium waste disposal were intensively discussed by the Nuclear Regulation Authority (NRA) in Japan, and a basic policy for near-surface disposal of uranium waste [17••] was presented by the NRA for public consultation. The study group worked as a platform for sharing relevant information and exchanging opinions about relevant governmental policies with the participants at study group meetings. In this review, we present the core information and summarize the major opinions shared in the study group from the viewpoint of intergenerational ethics.

## Current Situation of Uranium Waste Disposal

### Japan’s Policy for Uranium Waste Disposal

In Japan, the total amount of uranium waste is predicted to reach about 110,000 t (1.1 × 10^11^ g) by 2050 [25]. Those are a mixture of different components from a regulatory viewpoint: radioactive waste, industrial waste, and reusable materials after clearance; among them, approximately 50,000 t (about 20,000 bottles of 200-L drums) will be subject to burial disposal. Of these wastes, 84% will account for 1 Bq g^-1^ or less, 10 Bq g^-1^ or less for 93%, and 100 Bq g^-1^ for 98% [25]. Because these waste materials are temporarily stored as solid radioactive waste in the interim storage facilities on-site and some of the storage facilities are approaching capacity limits, prompt development and swift implementation of an appropriate disposal strategy are urgently needed.

**Fig. 1:**
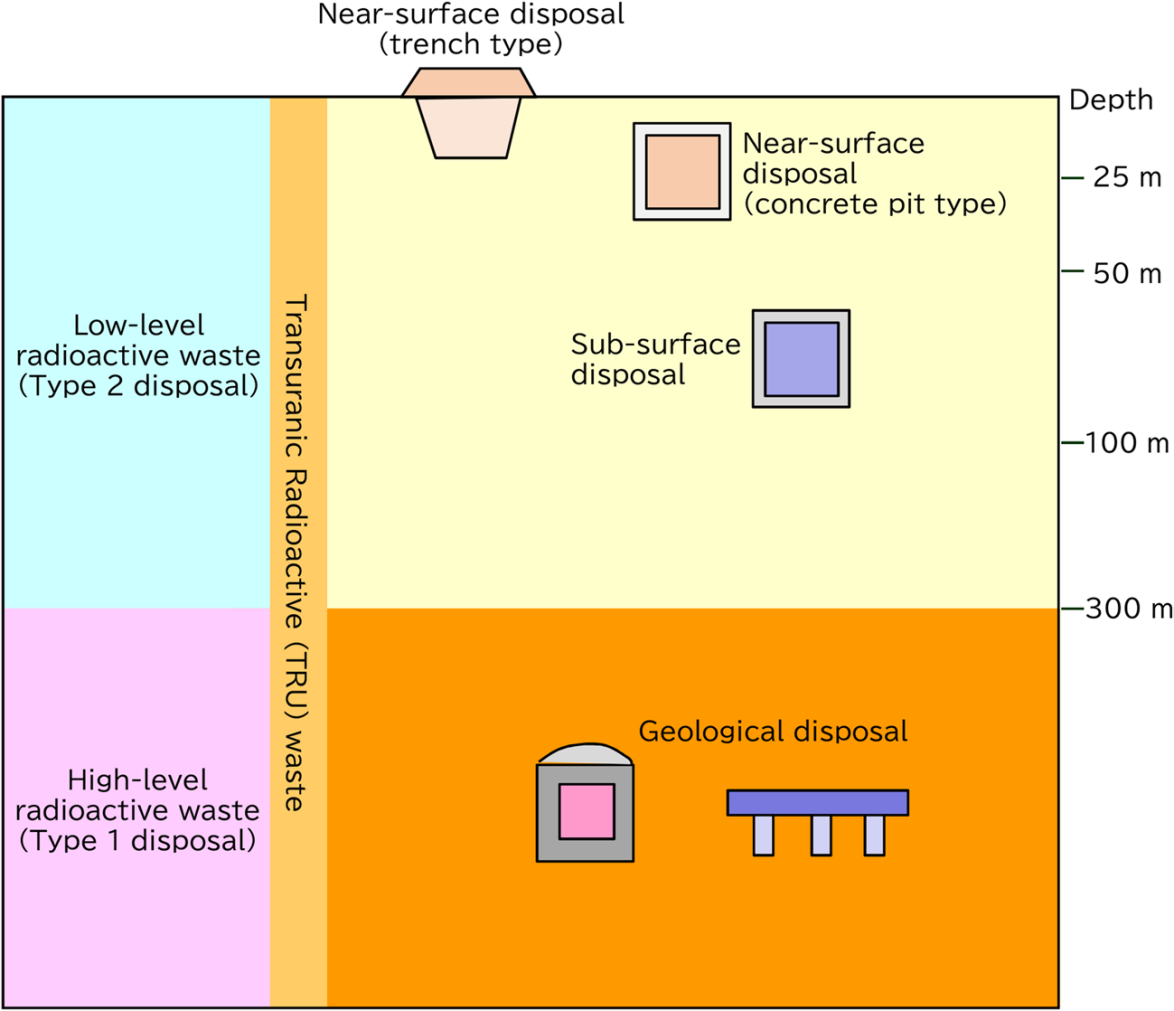
Methods of radioactive waste disposal; how to manage the uranium waste has been unclear for long time in Japan

Figure 1 shows a conceptual illustration of the types of radioactive waste disposal. There are two major methods for near-surface disposal: trench and pit. In subsurface disposal which is applied to the waste with relatively high radioactivity, the waste is buried deeper underground at a depth of 70 m or more from the surface. The near-surface disposal must meet the criteria for the impact assessment of buried waste on the public based on three typical scenarios: natural events, boring (making a hole with a drill), and assumed proximity of the public to the waste burial site. Geological disposal is the most realistic method for the final disposal of high-level radioactive waste. In this method, radioactive materials are isolated and confined for more than 100,000 years using an artificial barrier in underground stable bedrock at a depth exceeding 300 m.

In Japan, waste with a uranium concentration of less than 1 Bq g^-1^ (as a total of ^234^U, ^235^U, and ^238^U, which are naturally occurring parent uranium isotopes) can be reused or disposed of as general industrial waste if exempted from regulations based on the results of measurement and evaluation [26]. This clearance level is based on the fact that the average abundance of natural uranium in the earth’s crust is below 1 Bq g^-1^ [27]. Conversely, uranium waste with a concentration higher than this level (1 Bq g^-1^) is considered radioactive waste.

Meanwhile, owing to the unique feature that radioactivity does not decay over a long period as mentioned in the previous section, uranium waste had been treated in a different category from low-level waste derived from nuclear reactors and transuranic radioactive (TRU) waste containing transuranic elements, and policy on the disposal of uranium waste remained unclear. In recent years (~ 2021), the NRA of Japan held intensive deliberations on this matter and finally presented the basic concept of regulations regarding the clearance and disposal of uranium waste, where the NRA presented the regulatory concept for uranium waste clearance and burial as follows [17••]:

- Although it is appropriate to treat uranium waste as an artificial radioactive material, it is possible to consider that it has the characteristics of a naturally occurring radioactive material.
- The investigation should continue to bury uranium waste as a type 2 waste (low-level radioactive waste) under the condition that the initial uranium concentration is sufficiently low.

The former view is based on the idea that the ultimate goal of uranium waste disposal is to return uranium to nature as it originally existed. Regarding the latter view, for example, it is requested that the value of the total radioactivity amount divided by the total weight of the buried materials (uranium waste, artificial barriers, sand, etc.) should not exceed 1 Bq g^-1^, which is consistent with the condition in near-surface disposal with low-level radioactive waste. Uranium waste with a concentration exceeding this level (1 Bq g^-1^) must be disposed of either by subsurface or geological disposal.

The future generation of residents living close to a near-surface disposal facility can be exposed to buried radionuclides through several routes: external exposure, inhalation, skin contact, and ingestion through water and food. The Japanese authorities calculated the potential hazard of buried uranium waste that would bring a radiological risk to the people living directly above a near-surface disposal facility over hundreds of thousands of years [25, 26]. According to this prediction, the radioactivity level of buried uranium waste hardly change after several thousand years and will increase afterwards owing to an increase in the number of progeny radionuclides such as ^230^Th (half-life: 7.5 × 10^4^ years) and ^226^Ra (half-life: 1.6 × 10^3^ years). Consequently, in the case of 5% enriched uranium waste, the total radioactivity is expected to reach its highest level approximately 200,000 years after disposal.

**Fig. 2:**
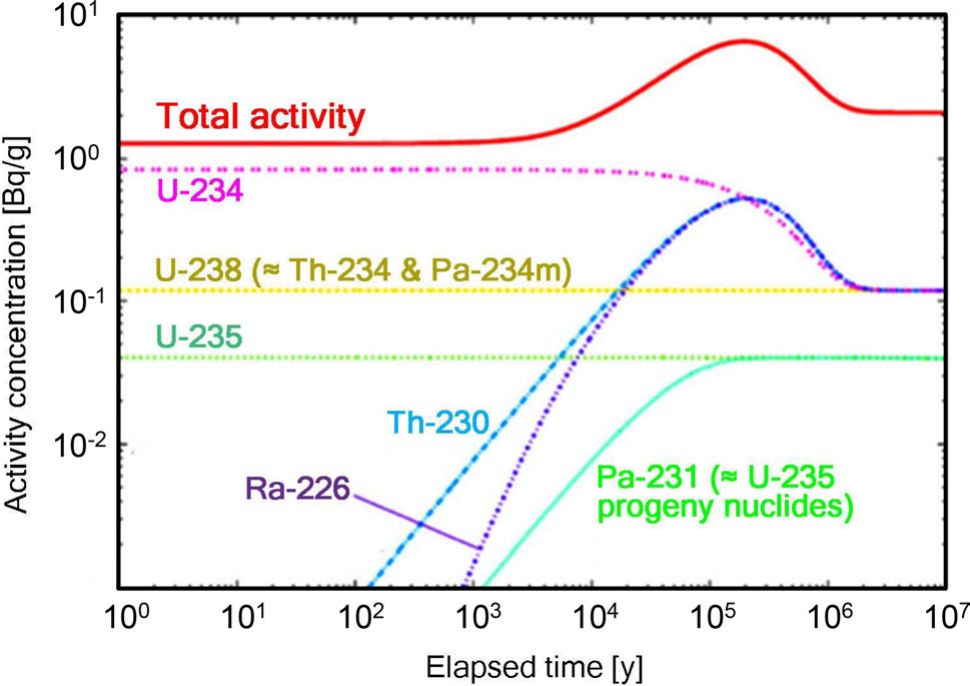
Predicted time changes of the radioactivities of major progeny radionuclides from 5% enriched uranium with 1 Bq g^-1^ of total initial concentration at the timing of disposal (reproduced from the calculations by the authorities in Japan [25, 26])

Among the progeny radionuclides, gaseous radionuclide ^222^Rn (half-life: 3.8 days, daughter of ^226^Ra) is difficult to contain, and the leaked ^222^Rn gas can cause internal exposure of residents living above or near the disposal facility through inhalation. Although the NRA predicted that the dose level owing to ^222^Rn would reach a peak approximately 200,000 years later (Fig. 2), the dose level varied significantly depending on the assumed situation, as shown in Table 2. When assuming that buried materials would not outflow from the disposal site to the external environment and that residents living above the waste burial site would directly inhale the leaked radon gas, the annual effective dose would be 5.9 mSv y^-1^ around 200,000 years later. However, with a conservative assumption of outflow, the dose would be 1.3 mSv y^-1^ at approximately 40,000 years. Although the maximum period for dose assessment of near-surface disposal of radioactive waste has often been set as 10,000 years [28], the potential risk of exposure beyond this period, that is, for a few hundred thousand years, is critical in the case of uranium waste disposal.

**Table 2:** Predicted peak annual effective doses and those timings of appearance from 5% enriched uranium when the total concentration of ^234^U, ^235^U, and ^238^U was 1 Bq g^-1^ at the time of disposal as low-level radioactive waste (reproduced from the results of calculations by NRA [17••])

	Predicted peak annual effective dose for different scenarios		
	Exposure from uranium and its progenies in the equilibrium	Exposure from progenies except radon	Exposure from radionuclides including radon
With conservative leakage	0.010 mSv y^-1^ (~ 1000 years after disposal)	0.18 mSv y^-1^ (40,000 years* after disposal)	1.3 mSv y^-1^ (40,000 years* after disposal)
Without conservative leakage		0.82 mSv y^-1^ (200,000 years* after disposal)	5.9 mSv y^-1^ (200,000 years* after disposal)

*Timing when the peak dose rate will appear

### Handling of Uncertainty in Super-Long-Term Evaluation

A predictive assessment for a super-long period of over 100,000 years involves extremely large uncertainties related to both natural phenomena and human activity. Accurately predicting the state of uranium waste buried underground, especially several meters deep near the surface, requires the evaluation of some sensitive but uncertain issues, such as the deterioration and alteration of the facility, gradual dilution of radionuclides due to leakage from the facility, and changes in the use of land including underground spaces. Even if it had the highest performance in terms of protective structures (e.g., thick soil cover and robust isolation walls), it is realistically foreseeable that any near-surface disposal facility would entirely lose its functionality 10,000 years later when the total radioactivity of uranium is reaching a peak (Fig. 2). Furthermore, the deterioration or loss of functionality would be highly accelerated owing to the effects of natural disasters such as earthquakes and torrential rainfall. Considering these unpredictable events, the IAEA states that the super-long-term safety of a radioactive waste disposal facility is not dependent on institutional control, whereas some credit for passive institutional control can be taken to prevent human intrusion during a certain period [29••].

Therefore, in the impact assessment of uranium waste disposal, it is necessary to guarantee that the resultant radiation exposure of distant future generations will be below the regulatory level, even when all protective measures of the facility lose their functionality. Therefore, it should be carefully considered that the potential risk of radiation exposure to residents would gradually increase because of the continuous generation of progeny radionuclides in accordance with the extremely slow decay of ^238^U and can exceed the current dose limit for members of the public (1 mSv y^-1^) [19] hundred thousand years later (Table 2).

**Fig. 3:**
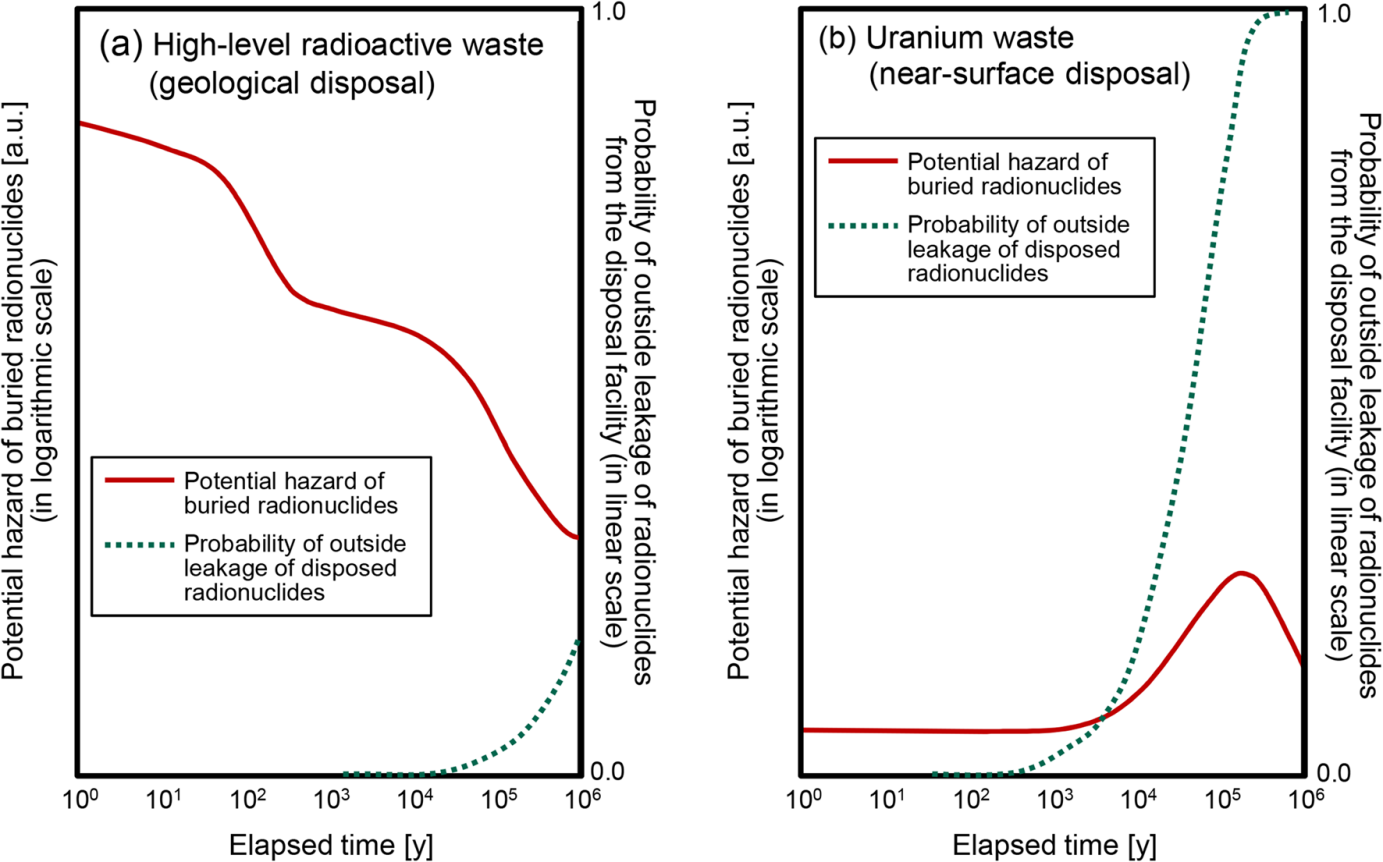
Comparative plots of predicted potential hazard (i.e., total radioactivity of the radionuclides concerned from the view of radiological protection) and probability of outside leakage of buried radionuclides from the disposal facility with regard to the cases of **a** geological disposal of high-level radioactive waste and **b** near-surface disposal of uranium waste. The hazard curves were reproduced from relevant references: **a** [30, 31] and **b** [25, 26]. The dotted curves of outside leakage probability were arbitrarily drawn based on the information on physical durability of each facility [31, 32]

As mentioned above, contrary to other radioactive wastes, the total radioactivity and potential risk of exposure to buried uranium waste have not been effectively reduced for hundreds of thousands of years. Figure 3 compares the predicted time changes of potential hazards (i.e., the total radioactivity of radionuclides concerned from the viewpoint of radiological protection) of buried waste and the probability of outside leakage of buried radionuclides from the disposal facility in cases of geological disposal of high-level radioactive waste (HLW) (Fig. 3a) and near-surface disposal of uranium waste (Fig. 3b). The temporary change curves of the hazard level were reproduced from relevant references: [30, 31] for Fig. 3a and [25, 26] for Fig. 3b. The curves of the probability of radioactive leakage from the disposal facility were arbitrarily drawn by the authors based on the information on the expected periods of hindering the mobilization of the radionuclides in the buried wastes: tens of thousands of years for the geological disposal facility and hundreds of years for the near-surface disposal facility [31, 32].

As seen in the figure, the potential hazard of buried HLW will significantly decrease after 100,000 years because the initial major components of HLW are fission products with relatively short half-lives, such as ^90^Sr (half-life: 28.8 years) and ^137^Cs (half-life: 30.1 years). Accordingly, the potential risk to future generations is expected to be significantly reduced, even after the protective measures of the disposal facility are lost. On the other hand, both the hazard level and potential risk of exposure to uranium waste are predicted to increase for hundreds of thousands of years, which implies that distant future generations have significantly greater risk than the current generation, regardless of protective measures.

Considering the difficulty in ensuring the durability of engineered barriers and also in predicting the situation of exposure through variable pathways (e.g., inhalation of dust, contact with skin, and entry through food and water), the IAEA stated that uncertainties in the long-term predictions limit the meaningfulness of the safety assessment in the case of near-surface disposal of long-lived radioactive waste. Therefore, the timescale for quantitative assessments may be limited, although they have noted that assessment time frames should be defined as appropriate for the possible changes in landscape and hydrological regime at the site [29••]. The NRA approach is in line with this statement, responding to the uncertainty of super-long-term assessments by confirming that the dose will not increase significantly after tens of thousands of years, even under highly conservative conditions regarding the effects of build-up and radon leakage [33]. For example, the assessment of the potential risk of radiation exposure assuming the loss of engineered barriers [25] and the reduction of the average uranium concentration in the entire disposal facility to below 1 Bq g^-1^ in near-surface disposal [26] can be interpreted as approaches to overcome the uncertainty accompanying the super-long-term assessment.

A point in the future projection of the NRA [17••] is that the leakage of buried materials outside the facility, whether intentional or not, significantly reduces the dose (Table 2). To date, discussions regarding the underground disposal of other low-level radioactive wastes have focused typically on achieving effective confinement for 1000 years based on the predictive assessment that the radioactivity of buried waste would largely decay for several hundred years, after which the radioactivity of the remaining long-lived radionuclides would gradually decline and reach the same level as that in the Earth’s crust. In such cases, a decline in the performance of protective measures (e.g., radiation shielding and radionuclide migration control) owing to facility deterioration is considered unfavorable. In contrast, in uranium waste disposal, where the ultimate goal is to return the waste to nature, leakage outside the facility at a slow pace could be preferable because it disperses the radionuclides in time and space and lowers the average radiation exposure of future generations. However, some residents living near the disposal facility may have anxieties about intentionally implementing measures to facilitate leakage and may judge it socially unacceptable.

## Ethical Arguments on Uranium Waste Disposal

### Consideration of the Benefits for Future Generations

In discussions on how to dispose of uranium waste, whose radioactivity has continued to increase over hundreds of thousands of years, the process of justification will become more important, as future generations could have different perceptions and scientific knowledge about radioactive waste from our own. Considering the responsibility of the current generation for respecting the dignity of future generations and ensuring their rights [22, 23], we need to answer the following questions to justify the uranium waste disposal that will cause potential harm in the distant future:

- Is it appropriate to treat current and distant future generations in the same way?
- Can we state that the benefits received by the current generation from the use of uranium are greater than the harm caused by uranium waste to future generations?
- Can waste disposal facilities bring any kind of benefit future generations?

In such discussions with distant future generations as imaginary stakeholders, we could not reach a single optimal solution but found many different solutions depending on assumed social changes and technological progress over the long term.

Judgment on justification, that is, considerations of benefits and harms for many stakeholders, has often been confused with utilitarianism, which is a way of thinking that emphasizes short-term economic rationality. Practically, benefits and harms include various tangible and intangible aspects, the cost-effectiveness of which cannot be determined easily. While it is commonly observed that the regulatory value of radioactivity level is first determined following a recommendation or guidance provided by the authority, and then exposures are managed to remain below the regulatory level in accordance with the principle of ALARA [19], this principle for optimization is preferably addressed in the discussion to determine the regulatory value of radioactivity. If we would probably lose a large benefit by taking measures to follow the regulatory level, it would be acceptable to change it flexibly, provided that radiological safety is ensured.

In the current thought of the Japanese authority [17••], it is unclear which exposure situation applies to the potential exposure that future generations could receive from buried uranium waste. Thus, it is unclear how the regulatory value of the radioactivity concentration (1 Bq g^-1^) [26] regarding uranium waste disposal was determined; specifically, whether it was based on a dose limit for planned exposure or a reference level for existing exposure. One reason for this value setting is that such a low concentration of uranium buried underground would have slight impact on people and the environment, even in the distant future, as the radioactivity concentration (1 Bq g^-1^) is comparable to the level of naturally occurring uranium in the Earth’s crust. In addition, it was inferred that regulation using radioactivity concentration poses a smaller uncertainty than regulation based on a predicted dose over a long-term period.

In particular, if radiation exposure from buried uranium waste is seen as planned exposure because the act of artificially burying radioactive materials underground is a human practice, the implementation of uranium waste disposal that could result in doses greater than the dose limit for the general public (i.e., 1 mSv y^-1^) would not be accepted. On the other hand, from the perspective of future generations, the exposure caused by radioactive materials buried in the past can be treated as existing exposure, because waste will already there when decisions for the management of radioactive materials are made. Under this circumstance, future generations may try to know the precise conditions of the disposal facility by themselves, reassess their safety, and place another regulation. For example, they may set a new regulatory value higher than 1 Bq g^-1^ for total radioactivity, including contributions from progeny radionuclides, based on the predicted doses of the general public of their time.

When the potential exposure of residents to the progeny radionuclides (especially ^222^Rn) of uranium buried near the surface becomes negligible in the distant future, it is probable that future generations with concerns about the health effects will decide to perform measures for risk mitigation, such as repairing the disposal facility or relocating uranium waste. This situation is caused by problems emerging on the facility side (e.g., deterioration of engineered barriers) as well as by changes in patterns in land use including underground space, the philosophical basis of radiological protection, and awareness of the health effects of buried materials among future generations. If the current generation tries to permanently dispose of uranium waste based on the current rules without considering the possibility of such changes over time, it could be seriously detrimental for future generations. Specifically, regarding the freedom and independence of future generations as a benefit for humanity, easy access to facilities and buried objects may become more important than the certainty of their confinement and isolation from the perspective of justification.

### Responsibility for Radiation Exposure of Future Generations

The current generation, which has benefited from the use of nuclear energy and is leaving radioactive waste to the next generation, has a responsibility to do their own best to increase the benefits and reduce the harm to them. In particular, in the case of uranium waste disposal, considerable efforts are required because the potential risk of radiation exposure caused by the progeny radionuclides will be higher in the distant future when the protective functions of disposal facilities will be completely lost. Regarding this intergenerational issue, ICRP has addressed “individuals and populations in the future should be afforded at least the same level of protection from actions taken today as is the current generation” [20••, 34]. Therefore, it would be inappropriate to provide a lower protection level for future generations owing to the unforeseeable situation in the distant future and to limit the assessment period to a shorter range (e.g., 1000 years) because of the difficulty of quantitative prediction.

As previously mentioned, the NRA of Japan considers 1 Bq g^-1^ as a sufficiently low radioactivity concentration and an acceptable regulatory value for uranium waste [17••], as uranium is a naturally occurring radionuclide that commonly exists at lower concentration in the earth’s crust. However, as shown in Table 2, even if the total radioactivity of primordial radionuclides, such as ^238^U and ^226^Ra was below 1 Bq g^-1^ at the time of implementing near-surface disposal, the dose to the general public could exceed the current dose limit (i.e., 1 mSv y^-1^) [19] ten thousand years later, mainly because of the contribution of gaseous ^222^Rn. If the protective performance of the disposal facility (shielding walls, soil cover, etc.) deteriorates faster than expected owing to unexpected environmental changes, the dose limit can be significantly exceeded.

While we can regard the exposure from buried uranium waste as existing exposure and avert the implementation of additional protective measures by setting a higher reference level (e.g., 10 mSv y^-1^), people of future generations may not support the idea that uranium waste produced by the past generation can be treated in the same way as naturally occurring radionuclides. For example, when high concentrations of ^222^Rn are detected near a disposal site in the future, people living there will become anxious and will try to investigate the cause of such an abnormal situation. After knowing the origin of their radiation exposure associated with considerable radiological risk, they might feel that they are unreasonably forced to bear unnecessary burdens.

It is noteworthy that the situation where a certain level of risk is socially present does not mean that it is socially acceptable [35]. In addition, in view of the fact that the system of radiological protection, including the definitions of doses and exposure situation categories have notably changed over the past few decades [18, 19, 36], it is highly probable that continuous, significant changes of basic concepts related to radioactive waste management would occur in the future. Moreover, the conceptual basis of the health effects of chronic radiation exposure at a low dose rate could significantly change with emerging scientific evidence in the near future [37, 38].

Considering these uncertainties, the current generation needs to decide and implement humbly a method of uranium waste disposal so that it could be continuously accepted by any generations. While dedicating our best efforts to find a disposal method to minimize the radiological risk posed to future generations, we need to recognize that the current generation cannot clearly know the harms and benefits for future generations.

### Communicating Information to Future Generations

People’s values and supporting knowledge bases have changed over time. There are inevitably large uncertainties in predicting how future generations will deal with waste containing uranium and how they will handle disposal facilities, as these uncertainties will be influenced by changes in society’s mainstream ideas and lifestyles. Looking back at history, we can say that any facilities that were well known by many people in the past gradually became neglected over generations and ended up in ruins owing to weathering, grave theft, and so on. Unless a system for information transmission is devised, the existence of an unvisited underground facility for waste disposal will get forgotten in a short period. It is possible that descendants who discover a neglected disposal facility in the distant future may not understand the purpose of its construction and may attempt to destroy the facility and extract its contents, that is, uranium waste.

To reduce such exposure of future generations due to ignorance, we need to ensure that they can get critical information regarding disposal facilities, including the types and radioactivity levels of radionuclides contained in buried materials, predicted radioactivity levels depending on elapsed time, structure and strength of disposal facilities, and methods to effectively reduce exposure from each nuclide. This information is required to transmit to the distant future in a robust and reliable manner.

In communicating with future generations, we should understand that the values of the majority of the current generation may not necessarily be supported by people in the distant future. Although it is acceptable to decide on a method of waste disposal based on the mainstream value of the current generation, it is inappropriate to conclude that one decision will be the best. What we should do is take the ideal method available at this time to dispose of uranium waste and transmit a relevant information to distant future generations. The information to be passed to the future can include the basic concept of radiological protection, the employed regulatory values (1 Bq g^-1^, 1 mSv y^-1^, etc.), the policy of “returning uranium to nature,” and the way of judgment for distinguishing between natural and artificial. To accurately convey information to the future, it is first necessary for the current generation to build consensus through wide-ranging discussions among people with a variety of ideas, with an emphasis on humanities and social science considerations.

Tondel and Lindahl [39••] indicated that it is necessary to convey information about the potential risk of radioactive waste using the concept of sustainable development in the distant future by referring to historical warnings carved on large stones in Sweden and Japan. They stated that even such epigraphic warnings intended as eternal messages had become ineffective in less than 2000 years and raised questions about how warning marks for a nuclear waste repository should be designed to last over millennia. While there are few cases where local residents are aware of the significance of the existence of older stone monuments, most of them have been removed or forgotten. In some cases, it seems that warning messages on graves rather encouraged destruction and grave theft. Based on these facts, we must admit that it is extremely difficult to transmit information to the future on a timescale of several thousand years. However, by using advanced modern technologies that are remarkably progressing at present, we could construct a novel effective communication system to overcome the difficulty of long-term, intergenerational information transmission.

The protection of future generations has also been discussed by ICRP. In Publ. 81 [34], the ICRP stated that ‘individuals and populations in the future should be afforded at least the same level of protection as the current generation.’ This view was succeeded in Publ. 122 [20••] which confirmed that the recommendation above continued to be valid and clearly stated that ‘the current generation has a duty of care to future generations.’ In Publ. 138 [21•] which presented four core ethical values underpinning the current radiological protection system: beneficence/non-maleficence, prudence, justice, and dignity, the view of intergenerational equity was indicated as an example of ‘justice’ with a statement that accountability in this context is part of implementing the value of intergenerational distributive justice.

The authors believe that the utmost efforts to share critical information with future generations for securing their safety are also linked to ‘prudence’ (underpinning precautionary measures) and ‘dignity’ (underpinning respect for autonomy). In light of these ethical values, the current generation needs to be fully aware that we are imposing the long-lasting potential risk of radiation exposure by disposing uranium waste and should fully utilize all available technologies to convey critical information about the burying materials to future generations, that is, to fulfill accountability to the people in the distant future.

## Conclusion

Investigations on uranium waste disposal in Japan have been conducted mainly from the perspective of natural science and engineering, based on predictions of radiological risks in typical scenarios, and the acceptability of the disposal plan has been evaluated in comparison with the limitation values of radioactivity or dose. Meanwhile, regarding the disposal of uranium waste, for which radioactivity cannot be expected to decay for a long period of up to several hundred thousand years, further investigation from the perspective of the humanities and social sciences is required. For example, we should consider the probable social positions of nuclear power utilization in future societies linked with the possible changes in ethical values and scientific knowledges that future generations could commonly have.

In light of human history, it is natural to assume that the present social systems and prevailing concepts underpinning our society will become unclear and no longer be understood by the next generation of people in thousands of years. It is probable that future generations will have different ideas about nuclear power utilization and plans to repair or dismantle existing facilities confining radioactive wastes in response to the deterioration in facility structure, changes in land use patterns, and new uses for uranium or progeny radionuclides. As the responsibility of the current generation, who have benefited from the use of nuclear power, we should not limit the benefits of future generations and leave options to handle disposal facilities flexibly according to their own will, while minimizing their potential radiological risk from the buried radioactive material. For achieving this, we should ensure that important relevant information regarding the disposal facility will be communicated to the distant future by utilizing the utmost out of the modern technologies.

The authors expect that the views presented in this article will be widely shared and further discussed by many stakeholders while hoping to contribute to resolving issues related to final disposal of radioactive waste.
